# Plant functional traits, but not soil abiotic properties, mediate the legacy effects of microplastics on plant community assembly

**DOI:** 10.3389/fpls.2026.1846448

**Published:** 2026-05-19

**Authors:** Xiu-Jing Ma, Wei-Ming He

**Affiliations:** College of Forestry, Hebei Agricultural University, Baoding, China

**Keywords:** legacy effects, plant community assembly, plant functional traits, soil microplastics, species richness, trait-mediated pathways

## Abstract

Soil microplastics (MPs) generate a persistent “memory” shaping the assembly of subsequent plant communities. While the direct effects of MPs have been extensively studied, the underlying pathways governing their legacy effects—whether they operate through altered soil properties (i.e., nutrients and pH) or modified plant traits—remain poorly understood. We examined MP legacy effects on 15 variables across a 12-level MP abundance gradient. Piecewise structural equation modeling (pSEM) was employed to quantify the relative contributions of soil abiotic properties and plant functional traits in mediating MP legacy effects on community assembly. MP legacy significantly increased grass abundance and soil nutrients, but decreased legume abundance. Across the abundance gradient, the majority of the 15 measured variables remained stable, with only the relative electron transport rate (rETR) showing a significant linear decline. pSEM revealed that the community-level shifts were not attributable to the changes in soil nutrients and pH. Rather, MP legacy effects were predominantly mediated via modifications to plant functional traits—specifically rETR, individual plant size, and root/shoot ratio—which emerged as the primary drivers of plant diversity, functional composition, and productivity. Consequently, the footprint of soil MPs on plant communities operates through a trait-mediated filter rather than the modification of soil abiotic properties. By identifying plant functional traits as the key mechanistic link, this study provides a framework for predicting the potential legacy consequences of soil MPs for plant community assembly.

## 1 Introduction

Microplastics (MPs) have emerged as a pervasive environmental challenge, with terrestrial soils increasingly recognized as a major sink for these persistent contaminants (de Souza MaChado et al., 2018; Thompson et al., 2024). Originating from diverse vectors—including agricultural film fragmentation, sewage sludge application, atmospheric deposition, and irrigation with contaminated water—MPs are accumulating in terrestrial ecosystems at an unprecedented rate (de Souza MaChado et al., 2019; Lozano and Rillig, 2022; Xiao et al., 2023). Recent surveys have detected MP abundances ranging from tens to thousands of particles per kilogram of dry soil across global landscapes (Chen et al., 2022; Xiao et al., 2023). In contrast to aquatic ecosystems where MP dynamics are often transient (Rillig et al., 2019; Baho et al., 2021), terrestrial ecosystems tend to retain these particles over decadal or even centurial timescales (Li et al., 2024; Augey et al., 2026). Due to their extraordinary environmental persistence, MPs exhibit half-lives spanning decades to centuries, depending on polymer identity and prevailing physicochemical conditions (Li et al., 2024; Augey et al., 2026). This longevity fosters a “soil MP legacy”—a sustained alteration of soil properties and biological functions that persists long after initial infiltration (Lozano and Rillig, 2022).

Despite a burgeoning body of research documenting MP impacts on soil properties (e.g., bulk density, water retention, aggregate stability, and nutrient cycling) and individual plant performance (de Souza MaChado et al., 2019; Wang et al., 2021; Zhang et al., 2025), several critical knowledge gaps remain. First, extant studies predominantly focus on direct exposure scenarios and individual-level responses (Baho et al., 2021; Zhang et al., 2022; Zantis et al., 2023), leaving the impacts of MP legacy on plant community-level attributes—such as species richness, functional composition, and primary productivity—largely unexplored (Zhang et al., 2025; Su et al., 2026). Compared to other global-change factors like warming and land-use change, the ecological footprint of MPs on plant communities remains understudied (Zhu et al., 2025). Second, current evidence stems largely from hypothesized, high-dose experimental scenarios, and there is a pressing need to evaluate responses across environmentally realistic gradients (MacLeod et al., 2021; Zhang et al., 2022; Zantis et al., 2023; Zhang et al., 2025). Third, existing experimental durations are often too brief (averaging 22 days) and biased towards agricultural cultivars, failing to capture the long-term ecological trajectories of wild plant communities (Zantis et al., 2023; Zhu et al., 2025).

The “legacy effects” paradigm posits that historical soil disturbances continue to orchestrate present ecological dynamics through indirect pathways (Cuddington, 2011; Zuppinger-Dingley et al., 2016; Heinen et al., 2020; Frouz, 2024). In MP-contaminated soils, these effects operate by modifying the habitat template—altering soil architecture, nutrient availability, and microbial symbiosis—which subsequently filters the recruitment and competitive ability of plant species (Lozano and Rillig, 2022). Crucially, plant-soil feedbacks—whether positive, negative, or neutral—mediate the divergent sensitivities of various plant functional groups to edaphic shifts (Reinhart and Callaway, 2006; van der Putten et al., 2016). Legumes are inherently dependent on microbial nitrogen (N) fixation, grasses are highly responsive to structural soil alterations, and forbs often display broader ecological plasticity (Wurst and Ohgushi, 2015; Nannipieri et al., 2023; Zhang et al., 2025). Such functional heterogeneity suggests that MP legacy is unlikely to exert uniform pressures across community attributes; rather, its impacts on diversity and productivity may be decoupled in both magnitude and direction.

Against this backdrop, our study addresses a pivotal question: To what extent does soil MP legacy restructure plant community assembly, and is this process governed by altered soil abiotic properties or modified plant functional traits? To test this, we implemented a high-resolution 12-level MP gradient within a two-phase experimental framework. We hypothesize that MP legacy acts as a selective ecological filter that favors specific functional strategies but suppresses others, thereby driving non-linear shifts in community assembly. By partitioning the relative contributions of soil physicochemical shifts and plant trait changes, this study provides a mechanistic understanding of how historical MP accumulation dictates the long-term trajectories of ecosystem functions. Consequently, our results provide a robust empirical foundation for defining sustainable soil health thresholds and predicting the impacts of MP accumulation on the resilience and functional stability of terrestrial ecosystems.

## 2 Materials and methods

### 2.1 Experimental design

**Figure 1:**
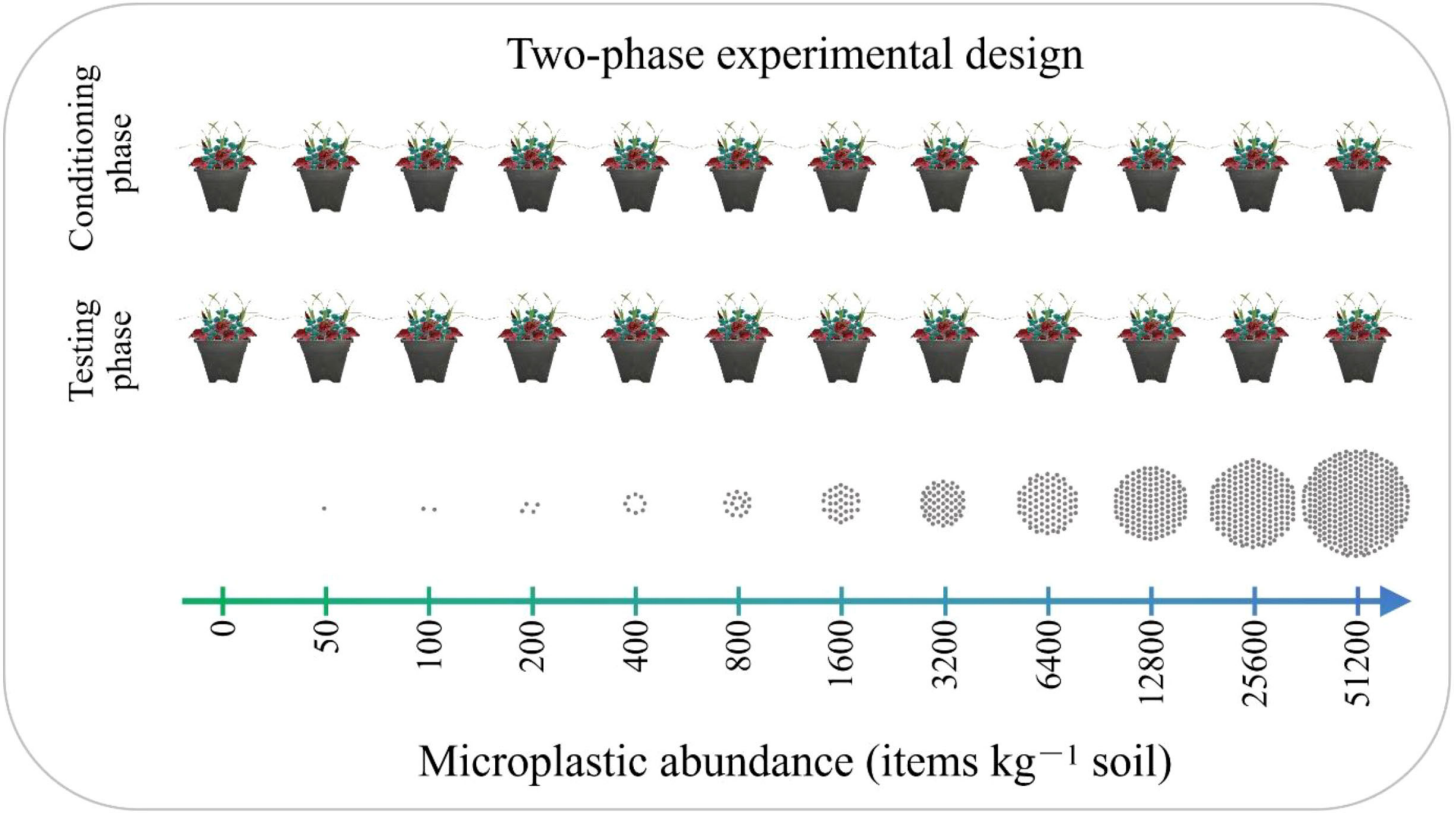
Schematic illustration of the two-phase experimental design, comprising a conditioning phase followed by a testing phase to assess legacy effects of MP exposure. The experiment was conducted across a 12-level MP abundance gradient (gray dots represent individual MP particles). At the onset of each phase, each mesocosm (a tapered square pot: top, 20 cm; bottom, 14 cm; height, 22 cm) was initialized with a standardized community of nine plant species. Eight replicate mesocosms were established per MP abundance level (*n* = 8).

The experiment employed a two-phase approach—comprising a conditioning phase (September 2023–January 2024) and a testing phase (February–June 2024)—to isolate the legacy effects of MPs (Brinkman et al., 2010). We utilized a full-factorial design crossing two phases with 12 MP abundance levels, resulting in 24 treatment combinations (**Figure 1**; a tapered square mesocosm: top, 20 cm; bottom, 14 cm; height, 22 cm), each replicated eight times (*n* = 192 total mesocosms). The MP treatments used fragmented polypropylene (PP; ~500 μm, white) sourced from Jiujuhe Plastic Company. PP was incorporated into a 1:1 (v/v) mixture of river sand and forest soil at 12 abundances: 0, 50, 100, 200, 400, 800, 1,600, 3,200, 6,400, 12,800, 25,600, and 51,200 items kg⁻¹ dry soil. This gradient encompasses environmentally realistic ranges (Chen et al., 2022; Xiao et al., 2023) as well as projected extreme scenarios. Furthermore, the gradient was specifically designed to facilitate abundance-effect analysis and to capture potential non-linear ecological responses (Hulme, 2008; Murren et al., 2014). The experimental soil is classified as cinnamon soil, the characteristic zonal soil type of northern China; it is characterized by a slightly alkaline pH (7.52–8.14) and moderate nutrient availability, with a mean organic matter content of 20.06 g kg⁻¹; the background abundance of PP MPs is relatively low, specifically below 200 items kg⁻¹ dry soil (Su et al., 2026).

We selected nine plant species with broad distributions as focal species: *Ageratina adenophora*, *Agropyron mongolicum*, *Elymus kamoji*, *Eupatorium odoratum*, *Lespedeza bicolor*, *Leymus chinensis*, *Onobrychis viciaefolia*, *Solidago canadensis*, and *Trifolium repens*. These species were further classified into three functional groups: forbs (*A. adenophora*, *E. odoratum*, and *S. canadensis*), grasses (*A. mongolicum*, *E. kamoji*, and *L. chinensis*), and legumes (*L. bicolor*, *O. viciaefolia*, and *T. repens*). Seeds of all nine study species were collected from the field by hand (for detailed procedures, see Su et al., 2026). It is noteworthy that grasses and legumes constitute the dominant plant species in local communities. However, these communities are susceptible to invasion by forbs, which possess the potential to colonize local plant communities due to their rapid proliferation and spread.

At the onset of each phase, we assembled a synthetic community by arranging all nine species in a 3 × 3 grid (one individual per cell) within each mesocosm, which served as an experimental analog to a natural grassland. To minimize spatial bias, species identities were randomized within their respective functional group positions across the grid. A sufficient quantity of seeds was sown at precisely designated coordinates within each mesocosm. Two weeks post-emergence, seedlings were gradually thinned to maintain only one individual per species, thereby establishing a standardized community density. Mesocosms were maintained in a controlled climate chamber (environmental parameters detailed in Zhang et al., 2025) and supplemented with water and Hoagland solution ad libitum. Specifically, a full-strength Hoagland solution was applied every three weeks. The solution contained macronutrients (1.0 NH_4_^+^-N, 14.0 NO_3_⁻-N, 1.0 P, 6.0 K, 4.0 Ca, 2.0 Mg, and 2.0 S mmol L⁻¹) and micronutrients (0.02 Cu, 2.8 Fe, 0.05 Zn, 0.5 Mn, 0.5 B, and 0.01 Mo mg L⁻¹). At the end of the conditioning phase, all plants were harvested. The testing phase immediately followed using the conditioned substrate and the identical sowing layout. Throughout the experiment, species richness and relative abundance were monitored, reflecting the integrated outcomes of survival and competition under MP stress.

### 2.2 Data collection

We focused on plant community properties—including plant species richness, plant community productivity, and species relative abundance—as well as potential mediators encompassing soil properties, plant growth, and community-level fluorescence. Prior to each harvest, we measured chlorophyll fluorescence of the plant community alongside soil pH, soil available N, soil available phosphorus (P), and soil available potassium (K).

Community-level fluorescence characteristics were quantified with the PlantExplorer^Pro+^ and its Data Analysis Package, as these metrics provide reliable indicators of the potential for plant communities to produce biomass and withstand abiotic stress. The fluorescence parameters measured included the chlorophyll index, relative electron transport rate (rETR), non-photochemical quenching (NPQ), and normalized difference vegetation index (NDVI). Soil pH and nutrient availability are known to be sensitive to MP addition and may serve as key mediators of plant and community performance (Zhang et al., 2025). Detailed protocols for chlorophyll fluorescence measurements and soil pH, N, P, and K analyses are described in a previous study (Zhang et al., 2025).

At the end of each phase, 96 mesocosms were available per experiment, and all individuals within each mesocosm were harvested. For each mesocosm, shoots were harvested separately by species, followed by the collective harvest of roots from all species, as the root systems of different species were intertwined and could not be separated. All shoot and root material was thoroughly rinsed to remove soil and debris, oven-dried at 80 °C for 48 h, and subsequently weighed to determine dry biomass—yielding shoot biomass per species and total belowground biomass across all species.

### 2.3 Data analysis

In the present study, soil nutrients were calculated as the sum of available N, P, and K; plant species richness was defined as the number of extant species per community; community productivity was defined as the total biomass per community. From the harvested biomass, we derived the following metrics to characterize community structure and performance: (1) aboveground biomass: the sum of shoot biomass across all plant species; (2) total biomass: the sum of above- and below-ground biomass, serving as a proxy for community-level productivity; (3) root/shoot ratio: belowground biomass divided by aboveground biomass; (4) mean individual plant size: the total biomass divided by the observed species richness; and (5) relative abundance of functional groups: the proportion of aboveground biomass attributed to forbs, grasses, and legumes, respectively.

Our two-step analytical approach—first assessing the effects of MP presence on 15 metrics, followed by an evaluation of dose-response effects along an abundance gradient—was employed to address the following scientific questions: (1) To what extent does the soil MP legacy modulate subsequent soil nutrients and pH, as well as plant and community-level performance? and (2) How do varying MP abundances shape the response patterns and structural assembly of synthetic communities?

To quantify the overall effect of soil MP legacies, we employed a one-way analysis of variance (ANOVA) to assess the impact of MP presence on soil chemical properties (pH, N, P, and K) measured at the end of the conditioning phase, as well as on plant community responses recorded at the end of the testing phase. In this analytical framework, the control group (no MP addition) comprised eight biological replicates, while the MP-treated group consisted of 88 biological replicates pooled across all MP abundance levels. This design facilitated a comprehensive assessment of the overall MP effect, providing a statistically rigorous comparison between the absence and presence of MPs consistently across all response variables. Given the substantial variation in scale among the 15 response variables (ranging from < 1 to > 40) and their differing units, all data were log_10_-transformed (log_10_(x+1)) prior to the ANOVA. This transformation was applied to normalize the variance and to ensure comparability across all variables.

To further investigate how soil MP legacy varies with MP abundances, we employed one-way ANOVA to test the legacy effects on each metric across the abundance gradient. For each metric, we applied regression modeling (linear, nonlinear, or polynomial) tailored to its specific response pattern along the MP abundance gradient. Given the broad range of MP abundances (50–51,200 items kg⁻¹ dry soil), which were log_10_-transformed prior to analysis to ensure statistical robustness and improve homoscedasticity.

Piecewise structural equation modeling (pSEM) was employed to elucidate the pathways by which soil MP legacy influences plant diversity, community productivity, and relative species abundance. In our conceptual model, MP abundance was defined as an exogenous variable, while soil properties (nutrients and pH) and plant physiological/growth traits (fluorescence characteristics, individual size, and root/shoot ratio) were treated as potential mediators. To satisfy the assumptions of normality and linearity, MP abundance was log_10_-transformed prior to analysis. To reduce dimensionality and represent complex constructs, soil nutrient availability was characterized as a composite index (by the sum of available N, P, and K). The pSEM was implemented using the piecewiseSEM package (v2.3.0.2). Model goodness-of-fit was rigorously evaluated using Fisher’s *C* and its associated *P*-value. Paths were iteratively refined based on modification indices to optimize explanatory power. The pSEM and associated analyses were executed in R version 4.5.1 (R Core Team, 2025). Statistical analyses were performed using IBM SPSS Statistics (version 27) and Origin 2025b (OriginLab Corp.).

## 3 Results

### 3.1 Overview of soil MP legacy effects

**Figure 2:**
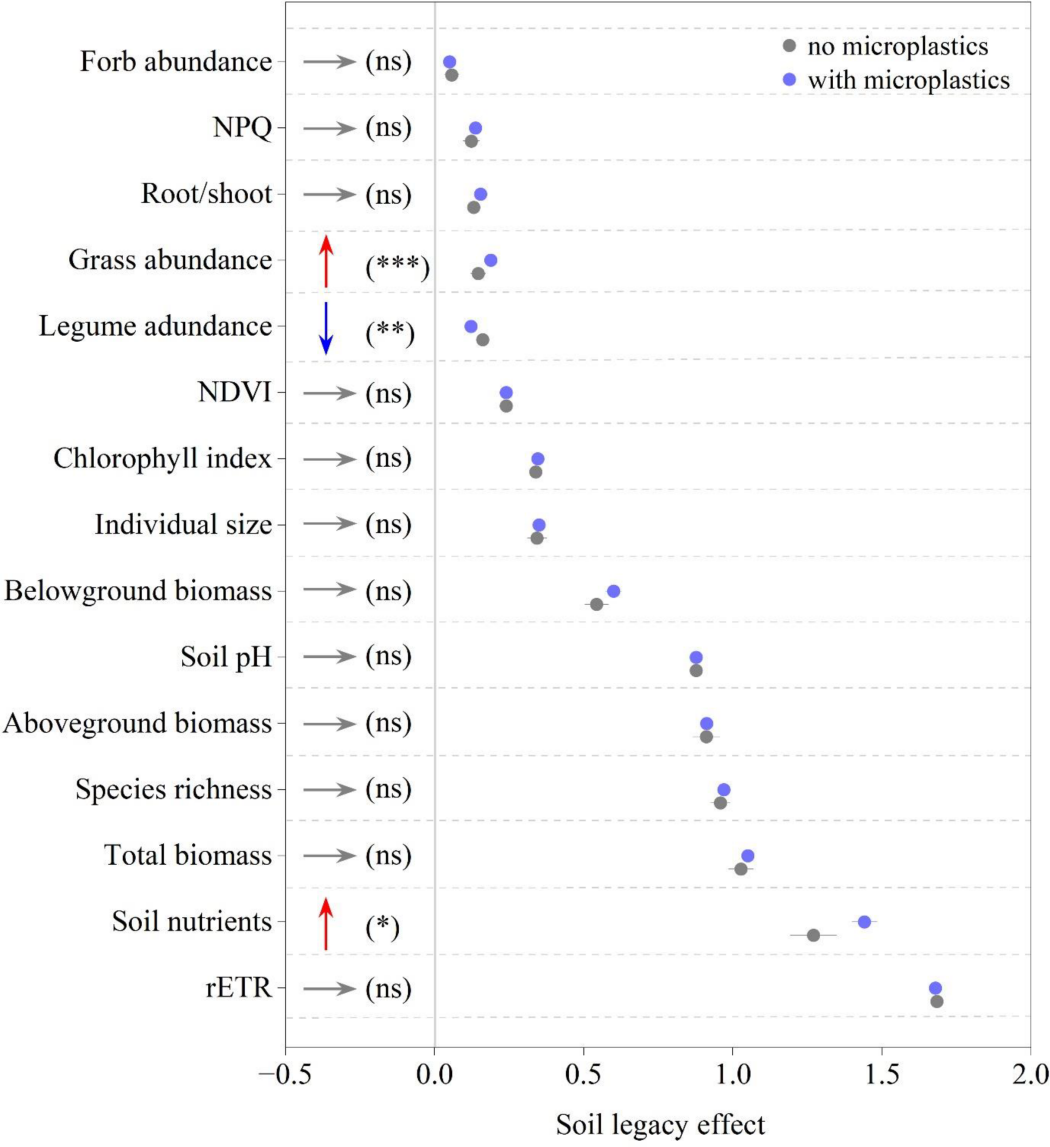
Soil legacy effects on 15 soil and plant variables. Gray and blue dots represent “no MPs” and “with MPs” treatments, respectively. Data points and error bars indicate mean values ± 2 SE (*n* = 8 for “no MPs”; *n* = 88 for “with MPs”). “ns” indicates that the presence of soil MPs fails to induce any significant legacy effects (*P* > 0.05); in contrast, asterisks denote significant MP-induced legacies at various confidence levels (*P < 0.05; **P < 0.01; ***P < 0.001). A red upward arrow signifies a significantly positive legacy from soil MPs, while a blue downward arrow represents a significantly negative legacy; horizontal gray arrows indicate no significant effects. NPQ, non-photochemical quenching; NDVI, normalized difference vegetation index; rETR, relative electron transport rate.

Overall, the soil MP legacy effects across the 15 measured variables exhibited a wide spectrum, ranging from adverse impacts to beneficial effects (**Figure 2**). When integrating across MP abundance levels, the presence of soil MPs failed to induce significant legacy effects for the majority of variables (*P* > 0.05). Specifically, significant differences between the “no MPs” and “with MPs” treatments were observed in only 20% (3 out of 15) of the variables (*P* < 0.05). Among these, the soil MP legacy significantly enhanced grass abundance and soil nutrient levels, indicated by the upward red arrows (*P* < 0.05), but significantly reduced legume abundance, indicated by the downward blue arrow (*P* < 0.01). For other variables, including biomass, chlorophyll fluorescence, and species richness, the legacy effects were neutral (indicated by horizontal gray arrows; all *P* > 0.05).

### 3.2 Responses along the MP abundance gradient

**Figure 3:**
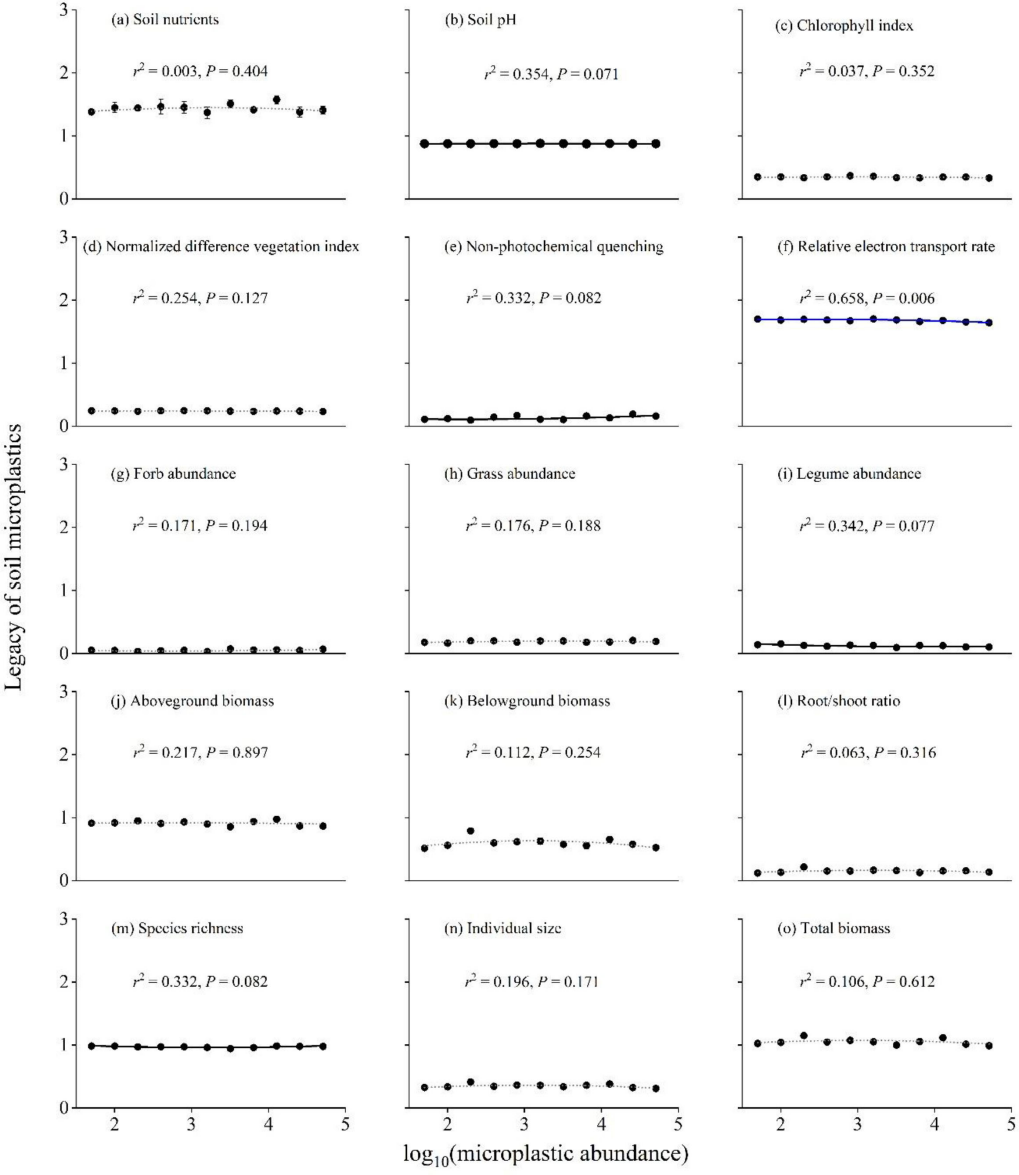
The relationship between log_10_-transformed soil MP abundance (x-axis) and the legacy effect of soil MPs (y-axis) on: **(a)** soil nutrients, **(b)** soil pH, **(c)** chlorophyll index, **(d)** normalized difference vegetation index, **(e)** non-photochemical quenching, **(f)** relative electron transport rate, **(g)** forb abundance, **(h)** grass abundance, **(i)** legume abundance, **(j)** aboveground biomass, **(k)** belowground biomass, **(l)** root/shoot ratio, **(m)** species richness, **(n)** individual size, and **(o)** total biomass. Data are means ± 1 SE (*n* = 8). Blue and black curves represent statistically significant (*P* < 0.05) and marginally significant (*P* < 0.10) regression fits, respectively, whereas dashed lines denote non-significant trends (*P* > 0.10). Coefficients of determination (*r*²) and associated *P*-values are shown in each panel.

Across the 15 variables, the legacy effects remained largely stable along the MP abundance gradient, with only one variable showing a statistically significant response (**Figures 3a–o**). Specifically, the relative electron transport rate (rETR) was the sole variable to exhibit a significant linear decline with increasing MP abundance (*r*^2^ = 0.658, *P* = 0.006; **Figure 3f**). In contrast, several variables showed marginally significant trends (0.05 < *P* < 0.10): soil pH (*P* = 0.071; **Figure 3b**), non-photochemical quenching (*P* = 0.082; **Figure 3e**), legume abundance (*P* = 0.077; **Figure 3i**), and species richness (*P* = 0.082; **Figure 3m**) all exhibited slight non-linear trajectories. However, for the majority of the indicators—including soil nutrients, chlorophyll index, NDVI, forb and grass abundances, and all biomass-related parameters—no significant functional relationships were detected (*P* > 0.10; **Figure 3**). These results suggest that within the tested abundance range, the soil MP legacy does not exert a consistent dose-dependent effect on most plant and soil properties.

### 3.3 Mechanistic pathways via pSEM

**Figure 4:**
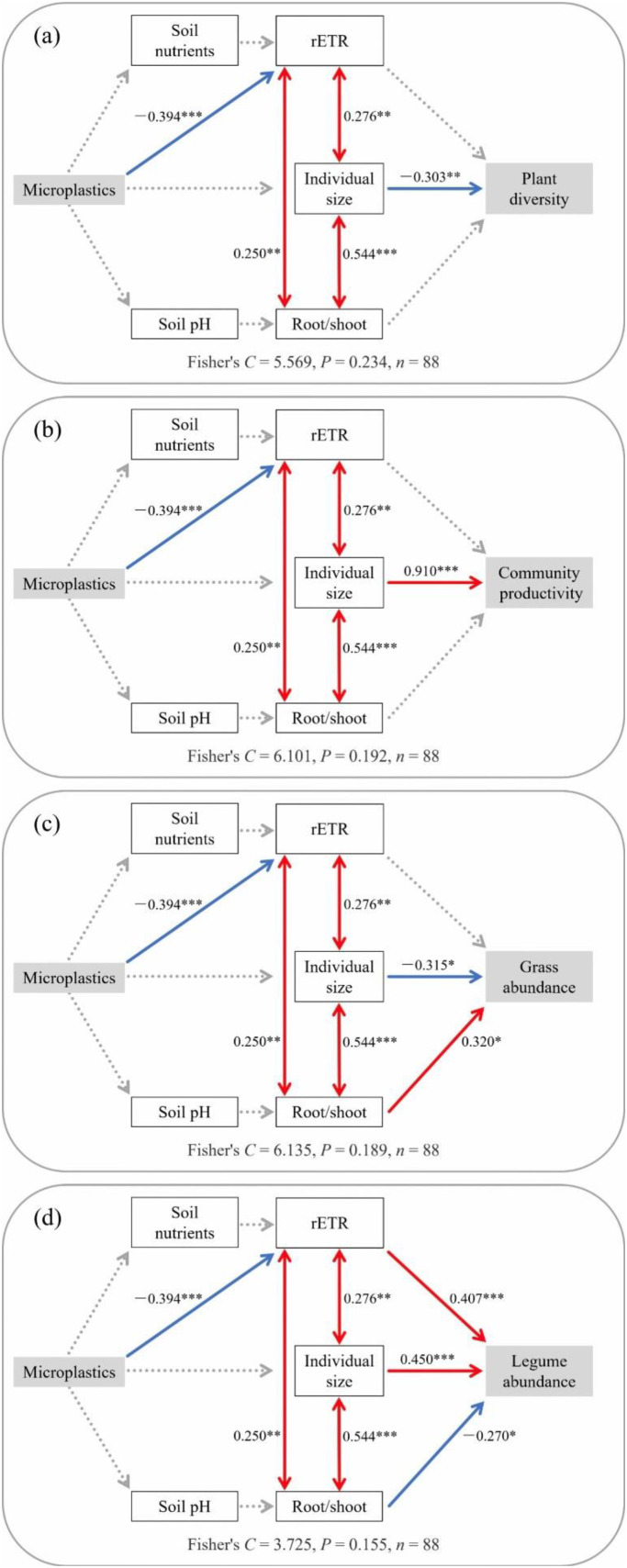
Piecewise structural equation models illustrating the pathways by which MP legacy influences plant diversity **(a)**, community productivity **(b)**, grass abundance, and legume abundance **(d)** through soil abiotic properties and plant functional traits. Single-headed arrows indicate hypothesized causal relationships, while double-headed arrows represent covariances. Red and blue arrows denote positive and negative significant paths, respectively; dashed arrows indicate non-significant paths. Significant level: *P < 0.05; **P < 0.01; ***P < 0.001.

The pSEM identified the specific pathways through which MP legacy effects shaped plant community attributes (Fisher’s *C* < 6.2, *P* > 0.05 for all models; **Figures 4a–d**). Across all models, MP legacy effects were primarily expressed through modifications to plant functional traits—specifically rETR, individual plant size, and root/shoot ratio—rather than through shifts in soil nutrients and pH. MPs consistently exerted a direct negative impact on rETR (*β* = –0.394, *P* < 0.001), which, alongside individual size and root/shoot ratio, acted as a key mediating pathway. These interconnected functional traits subsequently determined community-level responses, including diversity, productivity, and the abundances of grasses and legumes. In contrast, although pathways from MPs to soil nutrients and pH were included, these soil abiotic properties exerted no significant influence on plant traits or community assembly metrics, thus underscoring the predominant role of plant functional traits in mediating MP legacy effects.

Regarding plant diversity (**Figure 4a**), species richness was significantly suppressed by individual plant size (*β* = –0.303, *P* < 0.01). In contrast to diversity, community productivity was strongly enhanced by individual size (*β* = 0.910, *P* < 0.001; **Figure 4b**). For grass abundance (**Figure 4c**), root/shoot ratio had a significant positive effect (*β* = 0.320, *P* < 0.05), whereas individual plant size exerted significant negative effects (*β* = –0.315, *P* < 0.01). For legume abundance (**Figure 4d**), rETR (*β* = 0.407, *P* < 0.001) and individual plant size (*β* = 0.450, *P* < 0.001) emerged as the dominant positive driver, while the root/shoot ratio exerted a significant negative impact (*β* = –0.270, *P* < 0.05).

## 4 Discussion

### 4.1 Plant functional traits as the primary mediators of MP legacy effects

Our study provides compelling evidence that the legacy effects of soil MPs on plant community assembly are primarily mediated by shifts in plant functional traits rather than persistent alterations in soil abiotic properties. This pattern challenges the traditional “soil-centric” paradigm, which typically attributes MP impacts to altered bulk density, reduced water-holding capacity, or disrupted nutrient cycling (Rillig et al., 2019; Zantis et al., 2023; Zhang et al., 2025). Instead, our pSEM reveals a direct “trait-mediated filter” that overrides these abiotic factors. While MP conditioning did increase soil nutrient availability—likely due to inhibited root acquisition during the conditioning phase, as suggested by Su et al. (2026)—this increase exerted negligible explanatory power within the final model. Consequently, the ecological memory of soil MPs appears to restructure community assembly rules not by altering the soil nutrient pool, but by recalibrating the physiological responses and adaptive strategies of subsequent plants.

The shift toward a trait-centric perspective aligns with trait-filtering theory (Azeem et al., 2022; Grace, 2024), where MPs serve as a novel anthropogenic filter. Their physical presence may favor “acquisitive” functional traits—such as high specific leaf area or root length—capable of exploiting altered moisture and aeration patterns. This selective pressure leads to trait convergence among the surviving community members. Simultaneously, niche theory suggests that MP-induced disruptions to resource hotspots may shrink or shift realized niches, necessitating niche partitioning to minimize competitive overlap (Azeem et al., 2022; Grace, 2024). Consequently, community restructuring stems from a trait-based re-sorting of species in response to MP-conditioned soils.

The consistent negative effect of MP legacy on rETR, coupled with its significant association with individual plant size, points to a persistent physiological reprogramming. Specifically, the suppression of rETR in MP-conditioned soils suggests that historical exposure imprints a “stress footprint” on the photosynthetic apparatus (Baker, 2008; Lozano and Rillig, 2022; Pita-Barbosa et al., 2025). These physiological signatures indicate that plants grown in MP-legacy soils may engage in chronic photoprotective responses, likely driven by latent oxidative stress from the conditioned environment (Baker, 2008; Pita-Barbosa et al., 2025). Such effects appear embedded in the plants’ developmental trajectories, persisting well after the cessation of initial MP exposure (Ogle et al., 2015; Lozano and Rillig, 2022). This sustained physiological strain acts as a primary bottleneck, translating functional impairments into reduced individual vigor and, ultimately, diminished community-level productivity.

Collectively, these findings suggest that the legacy of soil MPs is encoded within the phenotypic variation of plants rather than being a simple reflection of altered soil properties. Given that soil abiotic properties failed to explain community restructuring in our model, we hypothesize that this ecological memory may be facilitated by persistent shifts in the soil microbiome. For instance, MP residues may selectively recruit microbial taxa that perturb plant hormonal pathways—such as ethylene or abscisic acid signaling—thereby affecting plant growth even in the absence of acute toxicity (Shi et al., 2023; Han et al., 2024; Pita-Barbosa et al., 2025). By demonstrating that MPs operate as a selective functional filter via trait-mediated pathways, this study provides a new framework for understanding how MPs encode a lasting biological legacy that shapes the successional trajectory and functional stability of terrestrial ecosystems.

### 4.2 Dose-independent stability and the “trigger” effect

Our results reveal that the relationship between MP legacy and plant community performance is largely stable across the abundance gradient, with the notable exception of rETR. Unlike the non-linear trajectories often observed in direct exposure studies (Zhang et al., 2025; Su et al., 2026), the legacy effects for the majority of indicators—including biomass and soil properties—did not exhibit significant dose-dependent responses within the tested range. This suggests that the “ecological memory” of MPs might be triggered at relatively low abundances, rapidly reaching a saturation plateau where further increases in MP abundance do not proportionally amplify the legacy impact. This finding deviates from the traditional ecological paradigm of dose-dependency (Calabrese and Baldwin, 2003; Hulme, 2008), suggesting that once the “memory” is imprinted via initial environmental conditioning, the magnitude of the subsequent community response becomes decoupled from the original pollutant concentration.

However, the linear decline in rETR with increasing MP abundance highlights a potential physiological tipping point. This specific sensitivity suggests that while the community’s physical structure (biomass) might appear resilient, its underlying metabolic vitality is progressively compromised as historical MP accumulation intensifies (Drechsler and Surun, 2018). The divergence between the stable response of most community indicators and the consistent linear decline of rETR prevents the identification of uniform abundance thresholds across variables. From a management perspective, this implies that the functional footprint of MP legacy is a pervasive feature of the ecosystem across a wide range of contamination levels; even in cases where plant productivity seems recovered, the “physiological memory” of MPs continues to exert a latent strain on plant performance.

### 4.3 Functional group sorting and the diversity-productivity decoupling

A key finding of this study is the differential sensitivity of plant functional groups to MP legacy, which drives a systematic restructuring of community functional composition. These patterns align with observations of the direct effects of soil MPs (Zhang et al., 2025; Su et al., 2026), confirming that primary functional groups—grasses, forbs, and legumes—are highly susceptible to alterations in the soil matrix (Yu and He, 2019; Lozano and Rillig, 2024; Zhang et al., 2025). While our selection of specific grass and legume species necessitates a cautious extrapolation to other taxa, the observed trends support the hypothesis that MP-conditioned soils act as a selective filter that suppresses legumes via trait-filtering and niche partitioning. Specifically, we propose the following hypotheses for future testing: (1) legumes will exhibit diminished nodulation and N-fixation efficiency in MP-legacy soils relative to other functional groups; and (2) MP residues in the soil matrix interfere with critical biochemical signaling—such as the flavonoid-nod factor exchange—between legume roots and rhizobia, ultimately undermining symbiotic success (Zahran, 1999; Han et al., 2024; Pita-Barbosa et al., 2025).

We also observed a notable structural shift: while the overall legacy effect of MPs on species richness and total biomass remained statistically neutral, their presence influenced plant functional dominance. This pattern suggests that by reducing the size of certain species—as evidence in the pSEM—MP legacy may alter competitive hierarchies. Specifically, the relative stability of species richness across the MP abundance gradient points to a competitive release (Crawley, 1997; Maestre et al., 2009). By reducing the size and dominance of fast-growing, resource-acquisitive species—reflected in the strong positive correlation between individual plant size and community productivity—MP legacy appears to diminish their capacity to monopolize light and space (le Roux and McGeoch, 2010; Spake et al., 2021). This “competitive leveling” allows subordinate and stress-tolerant species to establish and persist, thereby increasing alpha diversity even as the total functional output of the community does not increase (Crawley, 1997; Lima et al., 2022).

### 4.4 Study limitations and future research

Despite these mechanistic insights, our study is subject to three primary limitations. First, the use of a single synthetic community and polymer type (PP) under controlled conditions limits the extrapolation of our findings to natural ecosystems characterized by diverse plant functional groups and complex MP “cocktails” (e.g., varying polymers, sizes, and shapes). Second, while we identified a trait-mediated filter, the underlying mechanisms—specifically soil microbial succession and direct physiological indicators such as oxidative stress or photosynthetic rates—remain to be fully elucidated. Third, the temporal scale of this study was insufficient to determine the decadal persistence or evolution of these legacy effects across successive plant generations.

To build upon our findings, future research should focus on the following directions. First, studies must transition toward multi-factor field experiments that incorporate diverse MP types and complex plant communities to enhance the generalizability and predictive power of ecological risk assessments. Second, it is essential to unravel the mechanistic and transgenerational basis of observed trait shifts by integrating physiological measurements—such as oxidative stress and photosynthetic rates—and determining if these legacies are heritable via maternal effects or diminish over successive growth cycles in MP-free soils. Finally, investigating how trait-mediated MP legacies interact with global change drivers, such as climate warming and nitrogen deposition, will be critical for predicting the long-term trajectory and resilience of terrestrial ecosystems in an increasingly complex environmental landscape.

### 4.5 Concluding remarks

In conclusion, our findings indicate that the “ecological memory” of soil MPs is primarily expressed through a trait-mediated filtering process, rather than through alterations of the abiotic soil environment. Plant functional traits—particularly rETR, individual plant size, and root/shoot ratio—emerge as the dominant mediators of MP legacy effects. This legacy restructures community composition by favoring grasses while suppressing legumes, likely through the disruption of competitive hierarchies. Crucially, the legacy effect on most community attributes remained consistent across the MP gradient, suggesting a widespread functional shift that is triggered even at relatively low MP abundances. By identifying plant functional traits as the key mechanistic link, this study provides a framework for predicting the potential ecological footprints of soil MPs on plant community assembly and ecosystem stability.

## Data Availability

All the data of this study will be made available upon acceptance from the corresponding author on request.
